# Asymptomatic cryptococcal antigenemia is associated with mortality among HIV-positive patients in Indonesia

**DOI:** 10.7448/IAS.17.1.18821

**Published:** 2014-01-28

**Authors:** Ahmad Rizal Ganiem, Agnes Rengga Indrati, Rudi Wisaksana, Hinta Meijerink, Andre van der Ven, Bachti Alisjahbana, Reinout van Crevel

**Affiliations:** 1 Department of Neurology, Hasan Sadikin Hospital, Bandung, Indonesia; 2 Department of Clinical Pathology, Hasan Sadikin Hospital, Bandung, Indonesia; 3 Department of Internal Medicine, Hasan Sadikin Hospital, Bandung, Indonesia; 4 Department of Medicine, Radboud University Medical Centre, Nijmegen, The Netherlands

**Keywords:** cryptococcal antigenemia, meningitis, AIDS, antigen testing, lateral flow assay, Indonesia

## Abstract

**Introduction:**

Previous studies, mostly from Africa, have shown that serum cryptococcal antigenemia may precede the development of cryptococcal meningitis and early death among patients with advanced HIV infection. We examined cryptococcal antigenemia as a risk factor for HIV-associated mortality in Indonesia, which is experiencing a rapidly growing HIV epidemic.

**Methods:**

We included ART-naïve HIV patients with a CD4 cell count below 100 cells/μL and no signs of meningitis in an outpatient HIV clinic in Bandung, West Java, Indonesia. Baseline clinical data and follow-up were retrieved from a prospective database, and cryptococcal antigen was measured in stored serum samples using a semiquantitative lateral flow assay. Cox regression analysis was used to identify factors related to mortality.

**Results:**

Among 810 patients (median CD4 cell count 22), 58 (7.1%) had a positive cryptococcal antigen test with a median titre of 1:80 (range: 1:1 to 1:2560). Cryptococcal antigenemia at baseline was strongly associated with the development of cryptococcal meningitis and early death and loss to follow-up. After one year, both death (22.4% vs. 11.6%; *p*=0.016; adjusted HR 2.19; 95% CI 1.78–4.06) and the combined endpoint of death or loss to follow-up (67.2% vs. 40.4%; *p*<0.001; adjusted HR 1.57; 95% CI 1.12–2.20) were significantly higher among patients with a positive cryptococcal antigen test.

**Conclusions:**

Cryptococcal antigenemia is common and clinically relevant among patients with advanced HIV in this setting. Routine screening for cryptococcal antigen followed by lumbar puncture and pre-emptive antifungal treatment for those who are positive may help in reducing early mortality.

## Introduction

Cryptococcal meningitis is a major cause of subacute meningitis and death in patients with advanced HIV infection, affecting an estimated one million people each year, especially in sub-Saharan Africa [1, 2]. Cryptococcal meningitis is usually diagnosed by microscopic detection or culture of cryptococcus from cerebrospinal fluid (CSF), or detection of cryptococcal antigen in CSF or serum [3–5]. Serum cryptococcal antigen has also been found in HIV patients without clinical meningitis. In a cohort in South Africa, cryptococcal antigen screening adequately identified patients at risk for cryptococcal meningitis and death [6]. Therefore, pre-emptive antifungal treatment for patients with asymptomatic serum cryptococcal antigenemia has been advocated [7, 8]. Besides the South African study, several studies, most of them smaller, have been published from Uganda, Thailand and Cambodia [3, 9–13]. We report the largest cohort study so far, from Indonesia, which has one of the most rapidly growing HIV epidemics in Asia [14]. Most HIV patients in Indonesia present with advanced disease, and early mortality is very high [15]. We determined the prevalence of serum cryptococcal antigenemia and its effect on mortality among ART-naïve patients with a CD4 count below 100 cells/μL.

## Methods

### Setting and design

The study was conducted in Hasan Sadikin Hospital, the top referral hospital which serves the 42 million people of West Java Province, Indonesia. Since December 2004, integrated in- and outpatient services are provided for HIV-positive individuals. Services include HIV counselling and testing, HIV treatment and management of opportunistic infections. Since September 2007, patients are included into a cohort study and characterized prospectively using a standard questionnaire, physical examination and laboratory examination. Baseline blood samples are archived for all patients. Patients on ART generally visit the clinic monthly, with scheduled clinical and laboratory follow-up, including the measurement of CD4 cell count and plasma HIV-RNA [16]. Patients with symptoms suggesting central nervous system infection are referred to a neurologist for further investigations. CSF, if taken, is examined for *M. tuberculosis*, bacteria and cryptococcus [17]. Cryptococcal meningitis is diagnosed if cryptococcus is found in CSF, either with direct India ink staining or culture, or if cryptococcal antigen testing is positive. Patients diagnosed with cryptococcal meningitis are treated with either oral fluconazole or intravenous amphotericin B [17]. In-hospital death is recorded in medical records; outpatients are classified as dead if reported by family or community organizations or confirmed by telephone calls from the clinic. Patients not returning to the clinic are traced by telephone calls. No home visits are done as confidentiality might be breached since many patients do not disclose their HIV status to others. Baseline data and follow-up are recorded in an electronic database.

For this particular study, we included newly diagnosed HIV-positive adult patients presenting to the outpatient clinic between November 2007 and October 2011. Only ART-naïve patients with a CD4 count below 100 cells/μL were eligible for the study. Subjects with a diagnosis of cryptococcal meningitis on admission were excluded. Patient data needed for this study including sex, age, CD4 cell count, plasma HIV-RNA concentration (“viral load”), body mass index (BMI), baseline history of illness and follow-up were retrieved from a database of the HIV clinic. Tuberculosis (TB) at time of enrolment was defined as TB treatment started at some point between one month before and one month after baseline. The definition of cerebral toxoplasmosis was based on a physician's decision to start treatment with pyrimethamine and clindamycin for presumed cerebral toxoplasmosis. Oral candidiasis was diagnosed clinically. During follow-up, the time until initiation of ART was recorded, and clinical response was defined as alive, dead, lost to follow-up, or transferred out. Length of follow-up was counted from the day of inclusion until the date of death or the most recent date the patient was known to be alive. Patients were considered lost to follow-up if they had not returned to the HIV clinic within six months if ART-naive, or three months if already taking ART, and the last date they visited the clinic plus one day was considered the date of loss to follow-up.

### Ethics statement

Anonymous blood samples were retrieved from an already-existing hospital collection, collected as part of baseline laboratory assessment for patients included in the HIV cohort and approved by the Ethical Committee of Hasan Sadikin Hospital/Medical Faculty of Universitas Padjadjaran, Bandung, Indonesia (No. 114/FKUP-RSHS/KEPK/Kep./EC/2007). Informed consent had been asked prior to participation into the cohort, including consent to use archived samples; therefore, no separate consent was asked for this particular study.

### Laboratory examinations

Cryptococcal antigen testing was done using a lateral flow assay (LFA, Immuno-Mycologics, Norman, Oklahoma, USA), a recently developed and reliable point-of-care test approved by the US Food and Drug Administration [8]. For this study, serum cryptococcal antigen was measured retrospectively using serum samples archived at the time of enrolment in HIV-care (“baseline”). Quantification of the concentration of cryptococcal antigen in all positive samples was done using LFA according to the manufacturer's instruction. LFA titres were determined semiquantitatively by diluting samples in LFA diluent solution and assessing reactivity as described above. The highest sample dilution that produced a positive result as read by three observers was recorded as the LFA titre.

### Data analysis and statistics

Baseline characteristics of patients with and without cryptococcal antigenemia were compared. Continuous variables are presented as mean (standard deviation) if normally distributed and median (interquartile range, IQR) if not normally distributed, and categorical variables as percentage. Differences between groups were compared using Chi-square test for proportions and Mann-Whitney U test for continuous variables, with *p*<0.05 considered statistically significant. Progression to the primary endpoints death and a composite endpoint of death or loss to follow-up was examined by Kaplan–Meier estimates. Associations between cryptococcal antigenemia and these endpoints were examined using Cox regression. In multivariable Cox regression, we adjusted for possible confounding factors including CD4 cell count, haemoglobin, ART (as a time-dependent variable), oral candidiasis, toxoplasmosis and TB treatment, age and gender. Multiple imputation (five times imputation) was performed for time of death (as outcome), WHO clinical stage, and body height and weight, with assumption that all predictors were missing at random. All variables including time of death were used as predictors. Variables with *p*<0.100 in univariable analysis were included in the multivariable Cox regression model, and the final model was determined by backward selection. Statistical analysis was done by SPSS version 18.0.2 (IBM). The analysis presented used pooled data from the imputation, unless stated otherwise. Survival curves were drawn using GraphPad Prism version 5.0.

## Results

**Figure 1:**
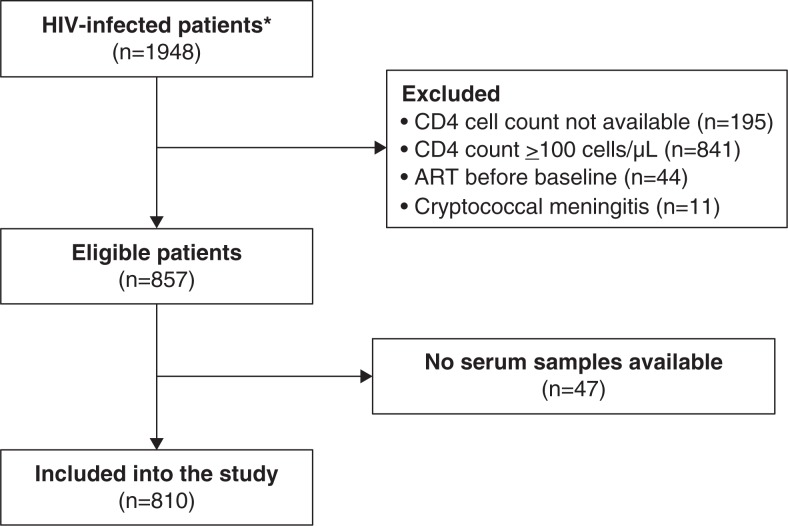
Patient flow. *All HIV-positive patients included in the HIV clinic cohort during the study period (2007–2011).

From 1948 patients registered in the HIV clinic during study period, 857 patients were eligible and 810 could be included in the study (Figure 1). Patients were mostly young males presenting with advanced disease and a very low CD4 cell count. Headache was reported by one of 266 patients for whom this information was available. Fifty-eight patients (7.1%) had a positive cryptococcal antigen test. There were no significant statistical differences in socio-demographic characteristics, clinical status, laboratory parameters, and symptoms at presentation between patients with and without serum cryptococcal antigen, except for WHO clinical staging as recorded in the medical records (Table 1). Fluconazole was used at time of inclusion by 120 out of 324 patients (37.0%) for whom this information was available. Fluconazole use was more common in patients who were found to have a positive cryptococcal antigen test (18/30; 60%), than in patients who tested negative (102/294; 35%). Titration of serum cryptococcal antigen using LFA showed a median titre of 1:80 (range: 1:1–1:2560). We found a weak correlation between CD4 cell count and the titre of cryptococcal antigen (*r*=−0.309, *p*=0.056).

**Table 1:** Baseline characteristics of patients according to cryptococcal antigenemia status^a^

	All patients (*n*=810)	CrAg-positive (*n*=58)	CrAg-negative (*n*=752)	*p*
Socio-demographics				
Age, year (IQR)	30 (27–34)	30 (25–32)	30 (27–34)	0.345
Male sex (%)	76.3	72.4	76.6	0.471
History of injecting drug use (%)	59.7	62.2	59.5	0.721
Clinical status				
WHO clinical stage 4 (%)	66.0	81.1	64.8	0.016
BMI<18.5 kg/m^2^ (%)	59.2	70.4	58.6	0.225
Laboratory parameters				
CD4 cells/μL, median (IQR)	20 (7–45)	18 (4–37)	21 (7–45)	0.327
HIV-RNA/mL, log (IQR)	5.2 (4.4–5.6)	5.0 (4.8–5.7)	5.2 (4.3–5.6)	0.923
Haemoglobin in g/dL, median (IQR)	11.3 (9.6–12.9)	11.1 (9.5–12.5)	11.4 (9.6–12.9)	0.322
Anaemia^b^ (%)	68.9	75.9	68.4	0.248
Positive HBsAg (%)	7.7	2.2	8.1	0.144
Positive anti-HCV (%)	60.7	59.6	60.7	0.875
Symptoms at presentation				
Fever (%)	43.9	44.8	43.8	0.884
Cough>1 week (%)	34.6	36.2	34.5	0.789
Weight loss>10% (%)	58.9	58.6	58.9	0.969
Co-infections at presentation				
TB treatment (%)	19.2	17.2	19.3	0.700
Cerebral toxoplasmosis treatment (%)	9.3	12.1	9.0	0.444
Oral candidiasis (%)	56.7	66.7	56.0	0.138

a Data were missing for TB treatment (*n*=1), haemoglobin (*n*=19), weight loss (*n*=20), cough (*n*=21), fever (*n*=22), oral candidiasis (*n*=82), history of IDU (*n*=88), WHO clinical stage (*n*=115), HBsAg (*n*=187), anti-HCV (*n*=195), BMI (*n*=271), HIV-RNA (*n*=720).

b Anaemia was defined as≤11.0 g/dL for females and≤13.0 g/dL for males. CrAg=cryptococcal antigen test.

Patients were followed for a median 343 (IQR 21–1000) days (total observation: 1190 patient years). A total of 561 patients (69.3%) started ART, after a median of 24 (IQR 14–42) days. After one year, 100 patients had died and 243 patients were lost to follow-up, corresponding with 12.3% one-year mortality and 30.0% one-year loss to follow-up. Death and loss to follow-up especially occurred during the first few months; 39 patients (39.0% of all deaths in one year) died within one month, and another 155 patients (63.8% of all patients lost to follow-up in one year) did not return after their initial visit.

During follow-up, six out of 58 patients (10.3%) with a positive cryptococcal antigen test were diagnosed with cryptococcal meningitis, while none of the 752 patients with a negative test did so. The cases of cryptococcal meningitis were diagnosed after a median of 91 days (range 17–219 days); three patients were diagnosed within three weeks after starting ART. Two out of six patients with confirmed cryptococcal meningitis died despite treatment with amphotericin B. The risk of developing cryptococcal meningitis was not associated with the cryptococcal antigen titre (median titre 1:80 (IQR 1:5–1:1216) for those who developed meningitis vs. 1:40 (IQR 1:6–1: 4480) for those who did not; *p*=0.813).

**Figure 2:**
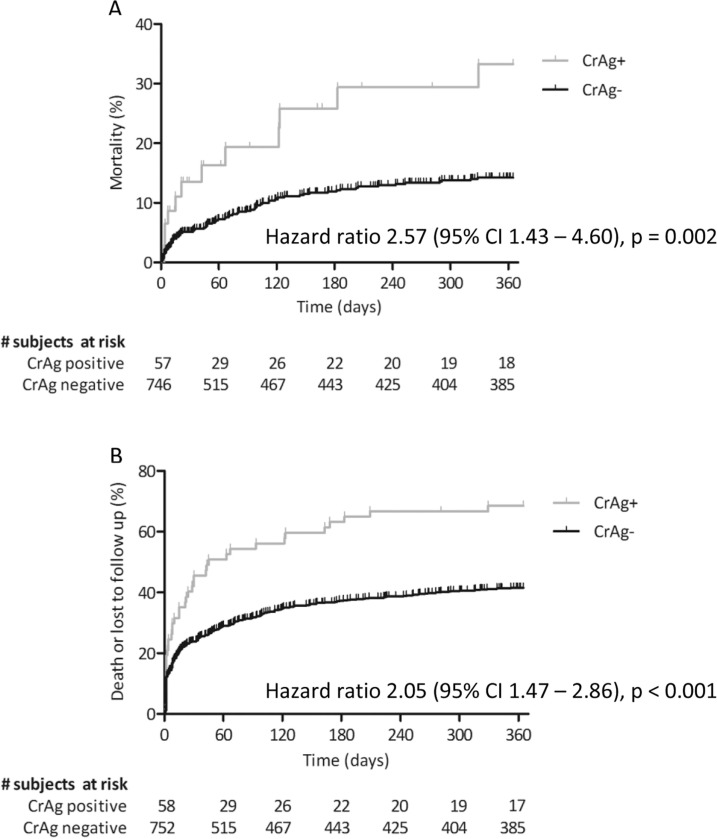
(A) Death in one year and (B) combined death or loss to follow-up in one year of subjects with and without cryptococcal antigenemia. CrAg+=positive cryptococcal antigen test; CrAg−=negative cryptococcal antigen test. Date of death was missing for seven patients (one CrAg+ and six CrAg−), who are not included in the survival curve in Figure 2A.

The date of death was missing for seven patients, for whom multiple imputations were performed. All variables had a similar distribution among those with missing data. A positive cryptococcal antigen test was clearly associated with increased mortality in one year, i.e. 22.4% vs. 11.6%, as also shown in the survival analysis in Figure 2A (HR 2.57, 95% CI 1.43–4.60; *p*=0.002). Mortality remained significantly higher among patients with a positive cryptococcal antigen test after two (22.4% vs. 11.7%) and three years (24.1% vs. 12.9%). Loss to follow-up was also higher among patients with a positive cryptococcal antigen test as compared to those with negative result (44.8% vs. 28.9%, *p*=0.011). It is likely that some patients who were lost to follow-up may actually have died, especially those who dropped out during the first few months. Indeed, more patients with a positive cryptococcal antigen test did not return after their initial visit to the clinic (29.3% vs. 22.5%), and more had reached the combined endpoint of death or loss to follow-up after one year (HR 2.05, 95% CI 1.47–2.86; *p*<0.001; Figure 2B).

Besides cryptococcal antigenemia, other factors associated with one-year mortality included ART administration, low CD4 cell count, anaemia at baseline, WHO clinical stage 4, BMI<18.5 kg/m^2^, a high plasma HIV-RNA and oral candidiasis. After correction for other factors, cryptococcal antigenemia remained significantly associated with one-year survival (Table 2) and the combined endpoint of death or loss to follow-up (Table 3).

**Table 2:** Factors associated with one-year mortality^a^

			Univariable^b^		Final model^c^	
			
	Dead (*n*=100)	Alive (*n*=467)	HR (95% CI)	*p*	HR (95% CI)	*p*
ART (time dependent)	–	–	0.39 (0.25–0.60)	<0.001	0.44 (0.27–0.71)	0.001
Positive cryptococcal Ag (%)	13.0	4.1	2.57 (1.43–4.60)	0.002	2.19 (1.78–4.06)	0.013
Male sex (%)	72.0	78.4	0.74 (0.48–1.15)	0.181	–	–
Age in years, median	31	30	1.02 (0.99–1.05)	0.261	–	–
CD4 cells/μL, median	11	23	0.98 (0.98–1.00)	0.004	–	–
WHO stage 4 (%)	82.0	58.4	2.81 (1.37–5.79)	0.007	2.28 (1.17–4.43)	0.017
HIV-RNA in log/mL, median	5.45	5.16	2.34 (1.21–4.53)	0.011	–	–
Anaemia^d^ (%)	83.0	63.5	2.59 (1.53–4.37)	<0.001	–	–
Oral candidiasis (%)	66.0	52.0	2.50 (1.54–4.05)	<0.001	2.26 (1.38–3.65)	0.001
Cerebral toxoplasmosis (%)	13.0	8.7	1.25 (0.93–1.69)	0.445	–	–
TB at admission (%)	17.0	19.4	0.76 (0.85–0.99)	0.300	–	–

a This table excludes those who were lost to follow-up. Cox regression was performed using pooled data from multiple imputations. Data were missing for anaemia (*n*=8), candidiasis (*n*=56), WHO clinical stage (*n*=115). BMI (*n*=115), HIV-RNA (*n*=473).

b Variables with *p*>0.100 were not included in multivariable analysis.

c HIV-RNA and BMI were not included in the final model because of the many missing values. Candidiasis was not included in the final model because of the possible interaction with WHO stage.

d Anaemia was defined as<11.0 g/dL for female and<13.0 g/dL for male.

**Table 3:** Cox regression analysis of composite end point death and loss to follow-up in one year^a^

			Univariable^b^		Final model^c^	
			
	Dead or lost to FU *n*=343	Alive *n*=467	HR (95% CI)	*p*	HR (95% CI)	*p*
ART (time dependent)	–	–	0.24 (0.19–0.31)	<0.001	0.24 (0.19–0.32)	<0.0001
Positive cryptococcal Ag (%)	11.4	4.1	2.05 (1.47–2.86)	<0.001	1.57 (1.12–2.20)	0.009
Age in year, median	30	30	1.01 (1.00–1.03)	0.084	–	–
Male sex (%)	78.2	78.4	0.81 (0.64–1.03)	0.090	0.75 (0.59–0.96)	0.022
CD4 cell count, median	15	23	0.99 (0.99–1.00)	<0.001	0.99 (0.99–1.00)	0.002
WHO stage 4 (%)	74.9	58.4	1.79 (1.27–2.51)	0.002	1.62 (1.16–2.28)	0.007
HIV-RNA in log/mL, median	5.39	5.08	1.78 (1.18–2.70)	0.006	–	–
Anaemia^d^ (%)	76.4	63.5	1.65 (1.28–2.13)	<0.001	1.46 (1.13–1.91)	0.004
BMI in kg/m^2^, median	17.19	18.08	1.49 (1.10–2.02)	0.011	–	–
Oral candidiasis (%)	63.3	52.0	1.41 (1.11–1.78)	0.004	–	–
Cerebral toxoplasmosis (%)	8.2	10.1	0.77 (0.64–0.94)	0.195	–	–
TB at admission (%)	14.6	22.5	0.65 (0.48–0.88)	0.005	0.61 (0.45–0.83)	0.002

a Cox regression was performed using pooled data from multiple imputations. Data were missing for anaemia (*n*=8), candidiasis (*n*=56), WHO clinical stage (*n*=115). BMI (*n*=115), HIV-RNA (*n*=473).

b Variables with *p*>0.100 were not included in multivariable analysis.

c Plasma HIV-RNA was not included in the final model because of the many missing values.

d Anaemia was defined as<11.0 g/dL for female and<13.0 g/dL for male.

ART was associated with a survival benefit. It reduced the risk of dying by 61% with every month of ART administration (*p*<0.001). CD4 cells count reduced the risk by 8.5% with every 10 cells increment (*p*<0.001). This benefit of having ART was significant for both cryptococcal antigen-positive and -negative cases, although the benefit was more prominent in the group of patients with cryptococcal antigenemia (risk reduction of 73.3% vs. 58.7%). Higher CD4 cells were only beneficial in those without cryptococcal antigen (risk reduction 9.1% with every 10 cells increment, *p*<0.001).

## Discussion

In this cohort of 810 patients with advanced HIV infection but with no clinical suspicion of meningitis, 7.1% had a positive serum cryptococcal antigen test. Those with a positive result had a much higher chance of developing cryptococcal meningitis (10.3% vs. 0%), and a higher chance of early loss to follow-up (44.8% vs. 28.9%) and one-year mortality (22.4% vs. 11.6%) as compared to those who had a negative result, also when corrected for possible confounding factors. To our knowledge, this is one of the largest cohorts of HIV patients examined so far and the first study from Indonesia.

The prevalence of serum cryptococcal antigenemia in our cohort was comparable to previously published studies that have screened HIV patients for circulating cryptococcal antigen in the absence of signs suggesting meningitis. In Uganda, cryptococcal antigenemia was found in 5.8% of 377 patients with a CD4 count below 100 cells/μL [9], in 8.8% of 295 similar patients from another study [10], and 19% in a recent cross-sectional survey among 367 hospitalized patients with a median CD4 count of 23 [13]. In South Africa, 7% of 707 patients screened were positive, including 12.5% of those with a CD4 count below 100 cells/μL [6]. A smaller study among 131 ART-naïve patients in Thailand found cryptococcal antigen in 9.2%, especially among those with a CD4 count below 100 cells/μL (*n*=85, 12.9%) [12]. Finally, in Cambodia, among a subgroup of 295 patients—most of whom had a CD4 count below 100 cells/μL but none of whom had signs of meningoencephalitis—10.8% were positive [11].

In our study, one in every 10 patients with a positive serum cryptococcal antigen test was diagnosed with cryptococcal meningitis during follow-up. In fact, this number may have been much higher as early loss to follow-up was very high and significantly associated with cryptococcal antigenemia. The majority of meningitis cases occurred after initiation of ART, possibly due to the “unmasking” effect of ART [18].

The presence of serum cryptococcal antigen was associated with at least a two-fold higher risk of death during follow-up, also after correction for possible confounding factors. Similar to the development of meningitis, our risk estimate for increased mortality may be an underestimate, as many cryptococcal antigen-positive patients who were classified as early loss to follow-up may in fact have developed severe cryptococcal meningitis leading to death. Mortality associated with cryptococcal antigenemia appeared lower, and seemed to occur somewhat later in our series (median time to death: 80 days) compared to a study in South Africa (median time to death: 53 days) and Uganda (median survival: 26 days) [3, 11], but this may have been biased by the high loss to follow-up in the first months in our study.

All patients in this cohort had an indication for ART, but 30% never started treatment, mostly because of early death or loss to follow-up. We were unable to retrace those patients who were lost to follow-up mainly because of fear of breaching confidentiality and stigmatization. However, in line with a systematic review and meta-analysis addressing loss to follow-up among HIV patients [19], it seems likely that many died.

This prevalence of cryptococcosis among asymptomatic patients found in our setting emphasizes the importance of screening patients with a low CD4 cell count for cryptococcal antigen, as has been advocated by experts in this field, and as was recently recommended by WHO [7, 8, 20, 21]. After completing this study we have implemented screening for cryptococcal antigen for all ART-naïve patients with a CD4 count below 100 cells/μL, followed by lumbar puncture for those with a positive result, and pre-emptive fluconazole treatment for those with a positive result but no meningitis. A recently published study from Uganda showed that primary prophylaxis of cryptococcal disease with fluconazole effectively prevented development of cryptococcal meningitis and cryptococcal-related death in HIV-positive individuals with no cryptococcal antigenemia, although all-cause mortality was not affected [22]. However, a targeted approach starting with cryptococcal antigen testing for patients with a CD4 count below 100 cells/μL prior to ART initiation may be more cost-effective than universal prophylaxis for all patients with low CD4 cell counts [10, 21, 22].

Our study suffers from several limitations. The outcome of patients who were lost to follow-up could not be verified. In addition, we may have missed cases of subclinical or mild cryptococcal meningitis at baseline. Since cryptococcal antigen testing was done retrospectively, no neurologist was consulted and no lumbar puncture was performed if there were no obvious signs of meningitis. On the other hand, as part of an ongoing cohort study on meningitis in our hospital [17, 23], physicians in our HIV clinic have a high vigilance for diagnosing meningitis. The study also suffers from the fact that fluconazole use (for possible other purposes) was not well recorded. Fluconazole, widely prescribed to patients presenting with advanced HIV infection in this setting, may have prevented the occurrence of overt meningitis and subsequent death. As such, our study may actually have underestimated the importance of cryptococcal antigenemia in this setting.

## Conclusions

Cryptococcal antigenemia was common and associated with at least 10% attributable burden of death and loss to follow-up in this large cohort of patients with advanced HIV in Indonesia. Despite the limitations of our study, this large cohort with long follow-up provides further evidence for the need to screen HIV patients with a low CD4 cell count for serum cryptococcal antigenemia, in an effort to reduce the high mortality of such patients. Future studies should be conducted to optimize screening and pre-emptive treatment of cryptococcosis.
